# Electrostrictive Mechanism of Nanostructure Formation at Solid Surfaces Irradiated by Femtosecond Laser Pulses

**DOI:** 10.1186/s11671-015-1224-5

**Published:** 2016-01-12

**Authors:** Oleg R. Pavlyniuk, Vitaly V. Datsyuk

**Affiliations:** Department of Physics, Taras Shevchenko National University of Kyiv, 64 Volodymyrska Str., Kyiv, 01601 Ukraine

**Keywords:** Electromagnetic forces, Laser-induced periodic surface structure, Surface plasmon polariton

## Abstract

The significance of the mechanical pressure of light in creation of laser-induced periodic surface structures (LIPSSs) is investigated. Distributions of the electrically induced normal pressure and tangential stress at the illuminated solid surface, as well as the field of volume electrostrictive forces, are calculated taking into account surface plasmon polariton (SPP) excitation. Based on these calculations, we predict surface destruction and structure formation due to inelastic deformations during single femtosecond pulses. The calculated fields of the electromagnetic forces are found to agree well with the experimental ripple structures. We thus conclude that the electrostrictive forces can explain the origin of the periodic ripple structures.

## Background

Femtosecond laser-pulse interaction with matter can lead to formation of laser-induced periodic surface structures [1–3]. Various applications of the laser-induced periodic surface structures (LIPSSs) have been proposed. They can be used for building microfluidic channels, controlling over laser marking and changing the color of materials [4, 5], overcoming the diffraction limit in laser nanomachining [6], modifying local electrical properties, improving the efficiency of solar cells [7], grating production, and optical data storage [8].

Though LIPSS was first observed in 1965 [9], the origin of the ripple structures is still debated [10]. Two types of basic formation mechanisms explained the observed structures. The first was a resonant mechanism based on (i) periodic electromagnetic-energy deposition due to roughness of the surface [11] or (ii) excitation of surface plasmon polaritons (SPPs) [1, 2, 6, 12–14]. The second non-resonant mechanism was related with thermal consequences of laser irradiation of the target, for example, capillary waves formed in the melted layer [15].

The classical resonant model [11] of the LIPSS formation assumed scattering of the incident laser wave by surface roughness and interference of the laser beam with refracted light. This interference causes inhomogeneous electromagnetic energy absorption just beneath the surface. According to this model, the period of the energy deposition can be smaller than the laser wavelength. However, initial roughness at the surface and several laser pulses are required to form LIPSS in this case [6]. In [16], the finite-difference time-domain method was applied to study the inhomogeneous energy absorption of a linearly polarized laser beam below the rough surface. The numerical results confirmed the resonant scenario.

A resonant mechanism for ripple formation involved excitation of the surface electromagnetic wave by the incident laser irradiation [1, 2, 6, 11–18]. According to [1, 6, 12, 14, 15, 17, 18], interference of the laser light with SPP creates periodic field distribution at the interface, which in some way leads to growth of a structure with the same period.

The SPPs can be excited at a metal surface. Therefore, LIPSS-covered metals were explained by SPP excitation [13, 14]. In addition, LIPSSs were observed at surfaces of semiconductors [1, 2, 12, 15, 17] and dielectrics [2, 6, 12]. Therefore, the possibilities and conditions of SPP excitation in semiconductor and dielectric materials were studied in [1, 2, 6, 12, 15].

Among the non-resonant mechanisms, it can be mentioned a theoretical model comprising heat transfer and hydrodynamic components [15]. The heat transfer component described particle dynamics, carrier excitation, and heat conduction phenomena. The hydrodynamic component described the molten material dynamics and the process of capillary wave solidification.

It should be noted that the timescale of the femtosecond laser pulses and SPPs (≃fs) is significantly less than the expected timescale of material movement (≃ps) [18] in the processes described above. Moreover, some authors [1, 17, 18] have demonstrated the possibility of the LIPSS creation just with a single femtosecond pulse. All these facts allow us to assume that mechanical action of the electromagnetic field can play an important role in the ripple formation. Therefore, the goal of our study is to determine the electromagnetic forces exerted on the material surface allowing for interference of the incident electromagnetic field with the excited SPP. We define a distribution of the electrically induced normal pressure and tangential stress at metal surface and estimate their amplitudes. In addition, we evaluate a density of the volume forces inside the surface layer. Then, we check correspondence between our calculations and known characteristics of the ripple structures.

## Methods

We applied the classical electromagnetic theory both to determine the properties of the SPPs and to calculate the force acting on a metal surface. For simplicity, we consider the interaction of a femtosecond laser pulse with a nonmagnetic metal (*μ*_2_=1 at *z*>0) in air (*ε*_1_=1, *μ*_1_=1 at *z*<0).

### Surface Plasmon Polaritons

In most cases, the ripples were observed to be perpendicular to the electric field vector [1–3, 6, 11–14]. Therefore, their origin was attributed to SPPs excited by a TM polarized mode [1, 3, 6, 7, 12, 14, 15, 17, 18].

**Fig. 1:**
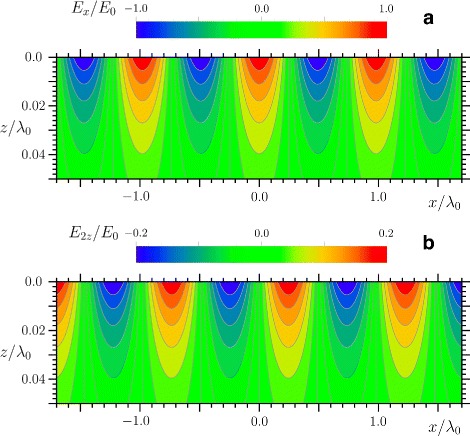
Electric field distribution of the SPP on copper surface. Calculated distribution of **a** *x*-component and **b** *z*-component of the electric field of the SPP excited at the air-copper interface at a frequency corresponding to a free-space wavelength of *λ*_0_=800 nm

The properties of the SPPs are described by the classical electromagnetic theory (see, for example, [19–21]). Namely, the fields that decrease exponentially with increasing distance from the metal-dielectric interface are found from the Maxwell’s equations (see Appendix 1 and Fig. 1). The dispersion relation for the SPP propagation constant *k*_p_ follows from the Maxwell’s boundary conditions giving

(1) $$ k_{\mathrm{p}} = k_{0}\,\left(\frac{\epsilon_{1}\epsilon_{2}}{\epsilon_{1}+\epsilon_{2}}\right)^{1/2},   $$

where *k*_0_=*ω*/*c* is the vacuum wave number, *c* the speed of light in vacuum, and *ε*_1_ and *ε*_2_ are the permittivities of the media at frequency *ω*.

According to Eq. 1 and conditions of SPP existence (one of dielectric constants is negative with an absolute value exceeding that of another) [19, 20], *k*_p_ is always larger than *k*_0_. Therefore, the SPP can be excited if there are nanoparticles at the surface and a localized plasmon excited around it [20]. The required nanoparticles can be created by electromagnetic forces acting on nanobubbles under the surface of metal [22].

### Maxwell Stress

A force acting on a dielectric body was first defined in Maxwell’s treatise on electricity and magnetism [23]. The time-averaged force can be expressed through the integral over a closed surface surrounding the body

(2) $$ \mathbf{F} = \oint \langle \mathbf{T} \rangle\cdot \mathbf{n}\,\mathrm{d}\/A,   $$

where **n** is the external unit vector normal to the surface, **T** the Maxwell stress tensor, and brackets denotes averaging over time. Among several forms of the Maxwell stress tensor, we chose one introduced by Lorentz

(3) $$ \mathbf{T} = \epsilon_{0}\,\mathbf{E}\otimes \mathbf{E} + \mu_{0}\,\mathbf{H}\otimes\mathbf{H}-\frac{1}{2}\left(\epsilon_{0}\, E^{2}+\mu_{0}\, H^{2} \right)\,\mathbf{I},   $$

where *ε*_0_,*μ*_0_ are the permittivity and permeability of vacuum, ⊗ denotes the outer product, and **I** is the unit matrix. The Maxwell-Lorentz tensor takes into account electrostriction and agrees with experiments better than other stress tensors [24].

Since the normal component of the electric field is discontinuous at the boundary between the media, the scalar product of **T** with **n** has a jump. The difference between 〈**T**〉·**n** in two media gives the surface force $\pmb {{\mathcal {F}}}$ which is measured in pascals. In order to relate the *z*-component of $\pmb {{\mathcal {F}}}$ with the normal pressure *P* on metal surface, we use the following definition:

(4) $$ \pmb{{\mathcal{F}}} = P\,\hat{\mathbf{z}}+\mathbf{S}= \left[ \langle \mathbf{T}^{(2)}\rangle -\langle \mathbf{T}^{(1)}\rangle\right]\cdot\hat{\mathbf{z}},   $$

where $\hat {\mathbf {z}}$ is a unit vector in the direction of the *z*-axis; the tangential component of $\pmb {\mathcal {F}}$ is called the tangential stress **S**. Formulas for calculating *P* and **S** are given in Appendix 2.

Application of the Gauss’ theorem transforms the right hand side of Eq. 2 into integral over the volume of the body

(5) $$ \mathbf{F} = \int \mathbf{\nabla} \cdot \langle \mathbf{T} \rangle \,\mathrm{d}\/V.   $$

The quantity under the integral sign is measured in newton per cubic meters and can be considered as the volume density of the electromagnetic force

(6) $$ \mathbf{f} = \mathbf{\nabla}\cdot\langle \mathbf{T} \rangle.   $$

## Results and Discussion

The normal pressure and tangential stress can be determined through the electric field **E** just beneath the surface (see Appendix 2). This field is a superposition of the transmitted electric field and the electric field of the surface plasmon wave **E**_2_. The normal pressure is expressed through the square of the normal component of **E** averaged over time. Here, we consider the incidence of a plane electromagnetic wave with the magnetic field parallel to the interface *z*=0, i.e., the case of p-polarization. In this case, the normal component of the electric field is nonzero and *E*_*n*_=*E*_*z*_. We neglect the reflected wave and took into account that the frequency *ω* of the incident wave is close to the plasmon frequency *ω*_s_ (usually, the central wavelength of the the femtosecond laser is at 800 nm). Under these assumptions, we obtain

(7) $$\begin{array}{@{}rcl@{}} \langle {E_{n}^{2}} \rangle \!\! & = & \!\! \frac{1}{2}\left(\left|\frac{\epsilon_{1}}{\epsilon_{2}}\right|^{2}\left|E_{\mathrm{\,i}z}\right|^{2} + \left|E_{2z}\right|^{2}\right)  \\ & &+\left|\frac{\epsilon_{1}}{\epsilon_{2}}\right| \left| E_{\mathrm{\,i}z}\right|\/\left|E_{2z}\right|\cos[(k_{\mathrm{p}} - k_{\mathrm{\,i}x})x],  \end{array} $$

where *E*_i*z*_ and *k*_i*x*_ are the *z*-component of the electric field and the *x* projection of the wavevector **k** of the incident wave, respectively; *E*_2*z*_ is the *z*-component of the SPP field defined by Eq. 19. Unlike the normal pressure, the tangential stress depends on both the tangential and normal components of the electric field. Therefore, we calculate

(8) $$\begin{array}{@{}rcl@{}} \langle E_{t}\,E_{n} \rangle \!\! &=& \!\! -\frac{1}{2}\left|\frac{\epsilon_{1}}{\epsilon_{2}}\right|\left|E_{\mathrm{\,i}x}\right|\/\left|E_{\mathrm{\,i}z}\right|  \\ & & +\, \frac{1}{2}\left|\frac{\epsilon_{1}}{\epsilon_{2}}\right|\left|E_{x}\right|\/\left|E_{\mathrm{\,i}z}\right|\sin[(k_{\mathrm{p}} - k_{\mathrm{\,i}x})x]  \\ & & +\, \frac{1}{2}\left|E_{\mathrm{\,i}x}\right|\left|E_{2z}\right|\cos[(k_{\mathrm{p}} - k_{\mathrm{\,i}x})x].  \end{array} $$

If *θ* is the incident angle, then *k*_i*x*_=*k*_0_ sin*θ* and

(9) $$ k_{\mathrm{p}} - k_{\mathrm{\,i}x} = \frac{2\pi}{\lambda_{0}}\,\left(\frac{\lambda_{0}}{\lambda_{\mathrm{s}}} - \sin \theta\right),   $$

where *λ*_0_=2*π*/*k*_0_ is the wavelength of the incident wave and

(10) $$ \lambda_{\mathrm{s}} = \lambda_{0}\left(\frac{\epsilon_{1}\epsilon_{2}}{\epsilon_{1} + \epsilon_{2}}\right)^{-1/2}  $$

is the wavelength of the SPP.

Equations 7–9 show that the period of the normal pressure and tangential stress is equal to

(11) $$ \Lambda = \frac{\lambda_{0}}{\frac{ \lambda_{0}}{\lambda_{\mathrm{s}}} \pm \sin \theta},   $$

where + corresponds to backward-propagating SPPs. The found periodicity is the same as the periodicity of the LIPSSs measured and estimated by a number of authors [6, 11, 12, 14, 15, 17, 18].

By using the formulas for *P* and *S* of Appendix 2 and Eqs. 7 and 8, we evaluated the magnitude of the surface force. All calculations were performed using the permittivity copper equal to *ε*_2_=−25.07+2.54 *i* at a wavelength of 800 nm. According to [1, 6, 15, 17], the amplitude of the electric field of the SPP can be of the order of the electric field of the incident wave. We took

(12) $$ E_{x}=E_{\mathrm{\,i}}   $$

and expressed *E*_i_ through the laser parameters

(13) $$ \frac{\Phi}{\tau} = \frac{\epsilon_{0}\,c}{2}\langle\left|\mathbf{E}_{\mathrm{\,i}}\right|^{2}\rangle,  $$

where *Φ* is the fluence of the laser pulses and *τ* is the pulse duration. Lasers with *τ*=140−150 fs, *λ*_0_=800 nm and *Φ* in a range of 0.2−1.5 J/cm^2^ are commonly used to generate LIPSSs. In numeric estimates, we used *τ*=150 fs and *Φ*=1 J/cm^2^ giving

(14) $$ P_{0}= \frac{\Phi}{\tau\,c}\simeq 0.2~\text{GPa}.  $$

Textbooks in optics identify *P*_0_ as the pressure of light on a perfectly absorbing medium with zero reflection coefficient [20]. If the reflection coefficient is equal to unity, the pressure would be equal to 2 *P*_0_. If *ε*_1_=1, light always pushes the absorbing medium exerting positive pressure, according to the common understanding of the radiation pressure. In this letter, we applied the Maxwell-Lorentz stress tensor to define the pressure *P* acting on a surface layer of a solid. In the case of normal incidence of light without plasmon excitation, one gets *P*=0 since *E*_*n*_=0. The normal component of the electric field is not zero if SPPs are generated. Using Eq. 12, we got *P*≃−12 *P*_0_ and a periodic *S*_*t*_ varying with a period *Λ*=*λ*_s_≃770 nm and amplitude 5 *P*_0_ at the surface of copper.

**Fig. 2:**
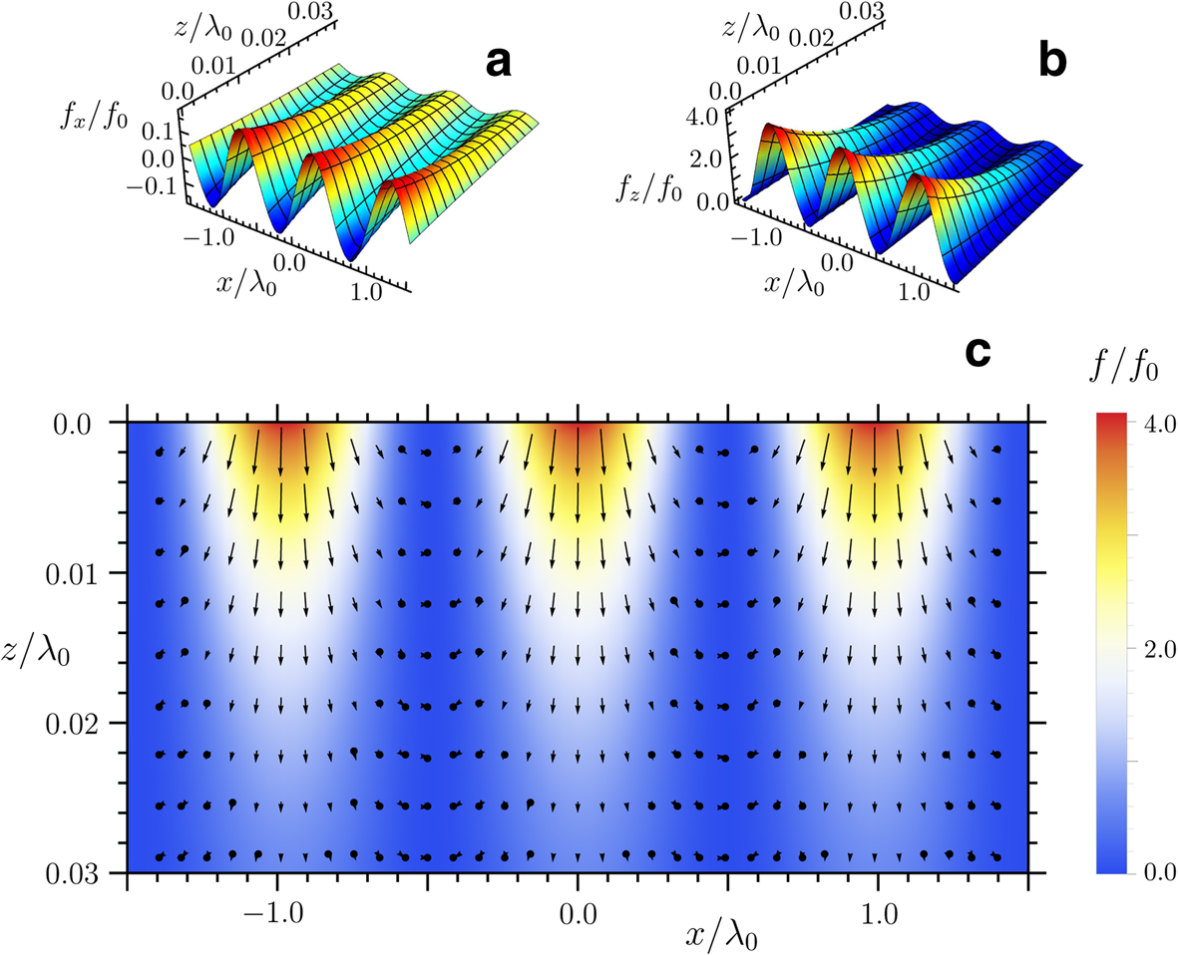
Distribution of the normalized volume electromagnetic forces inside copper. Calculated distribution of **a** *x*-component, **b** *z*-component, and **c** field of the volume force *f* inside copper in the case of normal incidence of a laser beam with the free-space wavelength *λ*_0_=800 nm

A picture of the light-matter interaction to be more complete if one calculates the volume force **f**. Thus, we found a distribution of **f** in copper for the case of normal incidence of a plane electromagnetic wave following by SPP excitation. The result of the calculation is presented in Fig. 2 which represents **f** in units of $f_{0}= \frac {1}{2}\, \epsilon _{0}\left |\mathbf {E}_{i}\right |^{2} \times 10^{9}$ m ^−1^. The integral $\int _{0}^{\infty } \mathbf {f}\,\mathrm {d}z$ gives as the pressure on a tin semi-infinite cylinder with the axis coincident with the *z* axis. The value of this pressure was found to be 50 *P*_0_. The volume force is distributed in a surface layer with a depth of about 25 nm. Our calculation thus show that the magnitudes of the surface and volume pressures can be as large as a few gigapascals. Such pressures should result in inelastic deformation of copper according to [25]. Figure 2 implies that a depth of the grooves created by a single femtosecond pulse could be about 25 nm.

We also established the period of the volume forces in the *x* direction. It is equal to 770 nm that is less than *λ*_0_=800 nm. The former number coincides with the period *λ*_s_ of the tangential stress found from Eq. 11 for the normally incident wave when *θ*=0.

## Conclusions

In this letter, we have proposed an electrostrictive mechanism of the LIPSS formation. We have calculated the electrically induced normal pressure and tangential stress exerted on the air-copper interface under interference of the incident laser field with the excited SPP. In addition, we determined the electrostrictive volume forces to a metal surface layer. The interfacial stress and the pressure on the surface layer have been found to reach a few gigapascals which is sufficient for copper surface modification. These calculations demonstrate that electromagnetic forces could be very important in the process of the LIPSS formation. The predicted period of the surface force distribution is in good agreement with the experimentally observed LIPSS periods and those given by other theoretical models. In the framework of the proposed theory, the depth of the grooves created with a single femtosecond laser shot has been also estimated.

## Appendices

### Appendix 1. Electric Field of Surface Plasmon Polariton

SPP is an electromagnetic excitation that may exist at the interface of two media with dielectric constants of opposite signs (for instance, dielectric and metal) [19, 20]. The electric and magnetic fields satisfy the Maxwell’s equations and ordinary Maxwell’s boundary conditions. The amplitudes of the fields decrease exponentially with increasing distance from the interface in both media. The electric field **E** has two components: *E*_*x*_ along the propagation direction of the wave and *E*_*z*_ perpendicular to the surface; the magnetic field **H** has only *y*-component.

The distribution of each component *A* of the electric and magnetic fields in a surface wave, propagating along the *x*-axis, has the following form:

(15) $$ A = A_{0}\,\exp(\pm k_{1,2}z)\,\exp[i(k_{\mathrm{p}}x-\omega_{\mathrm{s}} t)],   $$

where *A*_0_ is the amplitude, *k*_p_ is the propagation constant of the SPP, *k*_1_>0 and *k*_2_>0 are the damping factors of the surface electromagnetic wave in mediums 1 and 2, respectively, and *t* is the time. The upper “ + ” sign refers to medium 1 where *z*<0 and the lower “ −” sign refers to medium 2 where *z*>0. The coefficients *k*_1_ and *k*_2_ are defined as

(16) $$\begin{array}{@{}rcl@{}} k_{1} = k_{\mathrm{p}}\left(-\frac{\epsilon_{1}}{\epsilon_{2}}\right)^{1/2},\; k_{2} = k_{\mathrm{p}}\left(-\frac{\epsilon_{2}}{\epsilon_{1}}\right)^{1/2}. \end{array} $$

For a given amplitude *H* of the magnetic field, it is possible to express the amplitudes of the electric fields as follows:

(17) $$\begin{array}{@{}rcl@{}} E_{x} &=& \frac{i}{\left[-\epsilon_{1} - \epsilon_{2}\right]^{1/2}}\,Z_{0}\,H, \end{array} $$

(18) $$\begin{array}{@{}rcl@{}} E_{1z} &=&-\left[\frac{\epsilon_{2}}{\epsilon_{1}\,(\epsilon_{1} + \epsilon_{2})}\right]^{1/2}Z_{0}\,H,  \end{array} $$

(19) $$\begin{array}{@{}rcl@{}} E_{2z} &=&\left[\frac{\epsilon_{1}}{\epsilon_{2}\,(\epsilon_{1} + \epsilon_{2})}\right]^{1/2}Z_{0}\,H,  \end{array} $$

where *Z*_0_=(*μ*_0_/*ε*_0_)^1/2^.

### Appendix 2. Normal Pressure and Tangential Stress

The components *P* and **S** of the surface force $\pmb {{\mathcal {F}}}$ defined by Eq. 4 are readily found using the boundary conditions for the tangential components of **E** and **H** and normal components of **D** and **B**. Using the Maxwell-Lorentz stress tensor of Eq. 3, we obtain

(20) $$\begin{array}{@{}rcl@{}} P &=& \frac{\epsilon_{0}}{2} \left(1-\left|\frac{\epsilon_{2}}{\epsilon_{1}} \right|^{2} \right) \langle {E_{n}^{2}}\rangle +  \\ & & + \frac{\mu_{0}}{2}\left(1-\left|\frac{\mu_{2}}{\mu_{1}} \right|^{2} \right) \langle {H_{n}^{2}}\rangle,  \end{array} $$

(21) $$\begin{array}{@{}rcl@{}} S_{t} &=& \epsilon_{0} \left< \left(1-\frac{\epsilon_{2}}{\epsilon_{1}} \right) E_{t}\,E_{n}\right> +  \\ & & + \mu_{0} \left< \left(1-\frac{\mu_{2}}{\mu_{1}} \right) H_{t} \, H_{n}\right>.  \end{array} $$

In these equations, the the values of *P* and **S** are expressed through the electric field in medium 2. In the case of *μ*_1_=*μ*_2_, the surface force is independent of the magnetic field.
